# A multiplex liquid-chip assay based on Luminex xMAP technology for simultaneous detection of six common respiratory viruses

**DOI:** 10.18632/oncotarget.18533

**Published:** 2017-06-17

**Authors:** Yong Yan, Jian-Yong Luo, Yin Chen, Heng-Hui Wang, Guo-Ying Zhu, Pei-Yan He, Jin-Lei Guo, Yong-Liang Lei, Zhong-Wen Chen

**Affiliations:** ^1^ Jiaxing Key Laboratory of Pathogenic Microbiology, Jiaxing Municipal Center for Disease Control and Prevention, Jiaxing 314050, China; ^2^ Institute of Microbiology, Zhejiang Provincial Center for Disease Control and Prevention, Hangzhou 310051, China; ^3^ Lishui Municipal Center for Disease Control and Prevention, Lishui 323000, China

**Keywords:** Luminex, xMAP, respiratory virus, real-time RT-PCR, multiplex detection

## Abstract

We utilized one-step multiplex reverse transcription-PCR (RT-PCR) and Luminex xMAP technology to develop a respiratory multiplex liquid-chip assay (rMLA) for simultaneous detection of 6 common respiratory viruses, including influenza virus type A (FluA) and type B (FluB), para-influenza virus type 3 (PIV-3), respiratory syncytial virus (RSV), human metapneumovirus (MPV) and a threatening virus to China, Middle East Respiratory Syndrome coronavirus (MERS-CoV). Performance of rMLA was evaluated by comparing with real-time RT-PCR. Detection data from clinical specimens showed that the rMLA had diagnostic sensitivities of 97.10% for FluA, 94.59% for FluB, 98.68% for PIV-3, 94.87% for RSV and 95.92% for MPV (No Data for MERS-CoV due to the lack of positive specimens). Data of analytical sensitivities showed that the detection limits of the rMLA assay were 5–25 viral RNA copies per μl for FluA, FluB, PIV-3 and MERS-CoV, approximate to the real-time RT-PCR assay; while the values were 8 and 22copies/μl for MPV and RSV, lower than the real-time RT-PCR(78 and 114 copies/μl respectively). The results indicated that the rMLA is a sensitive, specific detection tool and comparable to real-time RT-PCR, especially suitable for high-throughput detection of respiratory specimens.

## INTRODUCTION

Human respiratory tract infection, mostly caused by respiratory viruses, gives rise to a considerable socio-economic burden in medical care and social productivity due to the significant morbidity [1–5]. Unfortunately, respiratory viruses are various and clinical symptoms are very similar, so that it is almost impossible to distinguish them clinically [6]. Therefore, a rapid, sensitive, specific and multi-target assay for detecting major and common respiratory viruses is desirable. In addition, rapid and accurate diagnosis is important for adopting early antiviral treatment, preventing nosocomial spread, decreasing stay duration and reducing patient management costs [7–9].

Traditionally, respiratory viral infections have been diagnosed by cell culture, rapid antigen/antibody test, or direct fluorescent assay [10, 11]. However, they are time-consuming, unable to give an early diagnosis. In many studies molecular diagnostic assays have been demonstrated to have superior sensitivity to conventional assays and are now being accepted as the new gold standard [12–15]. In particular, real-time PCR/RT-PCR shows significant superiorities due to its higher sensitivity and shorter turnaround time [16–19]. But, the limited multiplexing capacity of real-time PCR disables it to detect more targets and more clinical specimens in one assay simultaneously [12, 20]. The flexible multi-analyte profiling (xMAP) technology developed by Luminex Corporation, integrates flow cytometry, encoding microspheres, lasers and digital signal processing, and offers a molecular diagnostic platform for simultaneous, high-throughput and multiplex detection of up to 100 targets in protein or nucleic acid study. Now it has been approved by US FDA for clinical diagnosis [22] and is being used in various applications [20–25]. Several assays or kits based on xMAP technology have been developed for the nucleic acid detection of respiratory viruses [11, 20, 26–28], but they still need to be improved or exploited for the new emerging respiratory viruses, such as influenza A(H1N1 pandemic 2009) virus, avian influenza A(H7N9 2013) virus, Middle East Respiratory Syndrome coronavirus (MERS-CoV), etc.

In this study, one-step multiplex reverse transcription-PCR(RT-PCR) and Luminex xMAP technology were utilized to develop a respiratory multiplex LiquiChip assay (rMLA) for the detection of 6 common respiratory viruses in Jiaxing, including influenza virus type A (FluA) and B (FluB), para-influenza virus type 3 (PIV-3), respiratory syncytial virus (RSV) and human metapneumovirus (MPV), as well as a potentially threatening virus, MERS-CoV [29]. MERS-CoV was included into targets of the rMLA, considering the outbreak of MERS in South Korea in 2015 [30, 31] and the particular geographic location of Jiaxing, a north city of Zhejiang Province and near to Shanghai and Korea. The performance of rMLA was evaluated by comparing it to relevant real-time RT-PCR with clinical specimens from respiratory tract infection patients and synthetical standards from viral gene sequences.

## RESULTS

### Analysis of analytical performance

**Figure 1:**
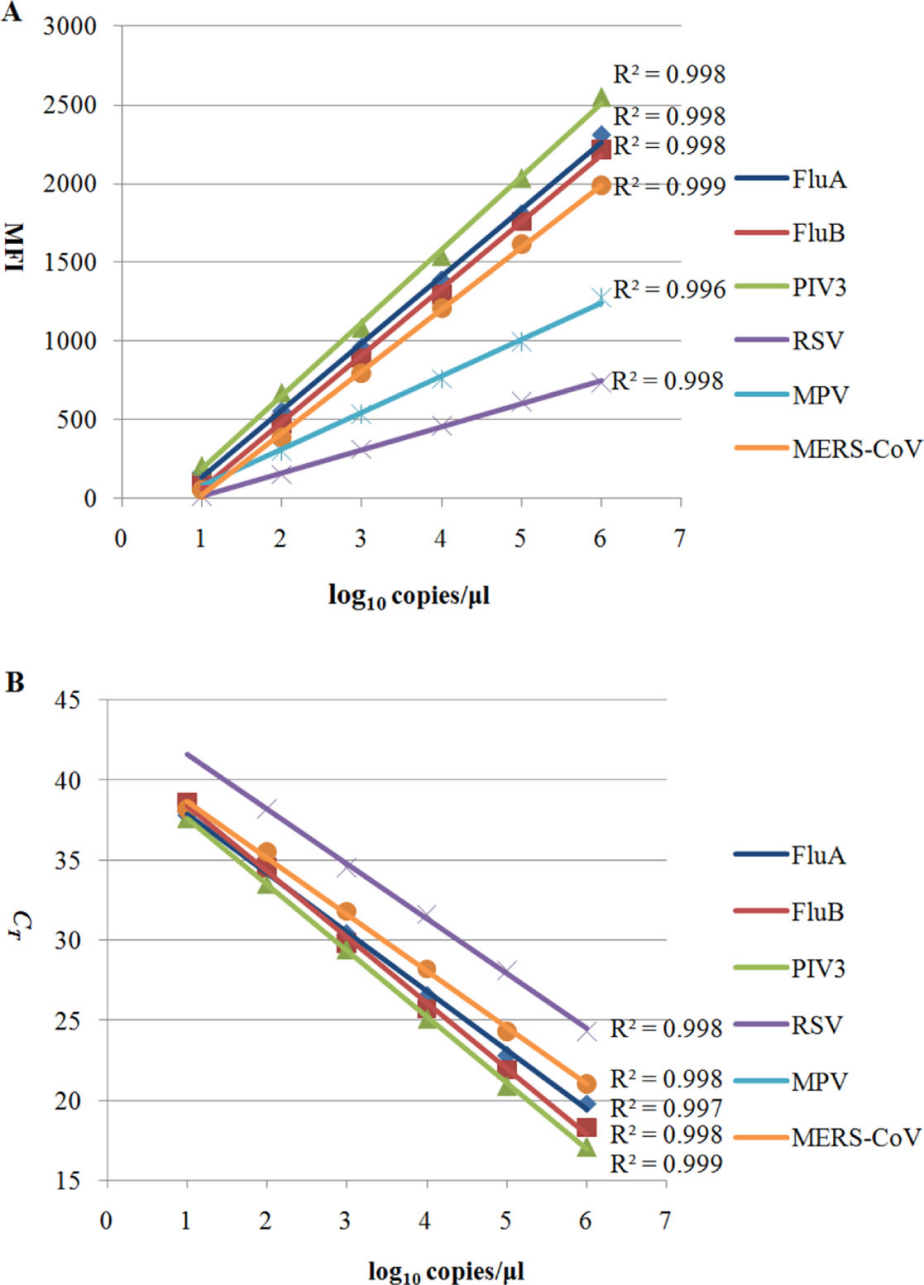
Comparison of the analytical sensitivity of the two assays developed in this study Viral *in vitro* RNA transcripts of six targets were used as standards. The concentrations of each target in standard curves were expressed in log_10_ copies/μl versus the median fluorescence intensity(MFI) values of Luminex-based rMLA assay (**A**) or the threshold cycle(*C*_*T*_) values of real-time RT-PCR assay (**B**).

The analytical sensitivities of the Luminex-based rMLA and multiplex real-time RT-PCR assay developed in this study were assessed by testing in duplicate 10-fold serial dilutions of positive standards ranging from 10^6^ to 10^1^ copies/μl of viral RNA transcripts for each target. The standard curves were drawn by the log_10_ values of standard concentrations versus the MFI values for rMLA (Figure 1A) and the *C*_T_ values for real-time RT-PCR (Figure 1B). By calculating from the curves at the pre-set cutoff value mentioned in MATERIALS AND METHODS, the detection limits of the two assays were determined to be 7, 10, 6, 22, 8, 11 and 9, 12, 8, 114, 78, 15 copies per μl for FluA, FluB, PIV-3, RSV, MPV, and MERS-CoV, respectively.

**Figure 2:**
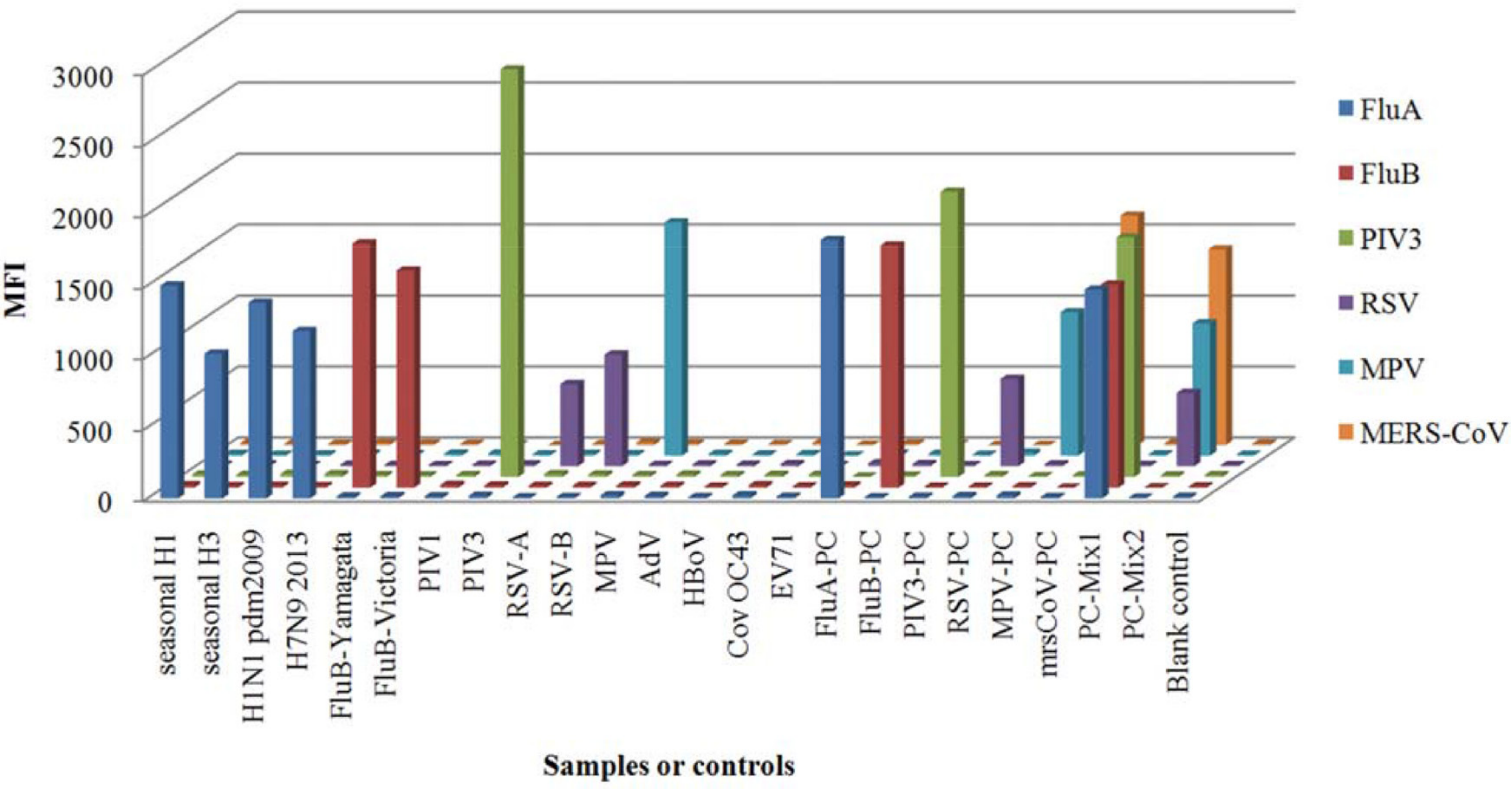
Analytical specificity of the Luminex-based rMLA assay The specificity analysis was carried out with viral RNA from positive samples of influenza A virus seasonal H1N1 and H3N2, H1N1 pdm 2009, H7N9 2013, influenza B virus Yamagata and Victoria lineage, PIV1, PIV3, RSV-A, RSV-B, MPV, AdV, HBov, Cov OC43, EV71, and positive controls(PC) of 10^5^ viral copies/μl for each target. Two random mixtures of positive controls, PC-Mix1 and PC-Mix2, were used to analyze multi-target detection capacity for co-infection samples briefly. Free-Rnase water was used as a blank control . Biotin-labelled RT-PCR products of samples or controls (*x* axis) were identified by bead-coupled capture probes (*z* axis) and showed by the MFI values (*y* axis).

The analytical performance of the two assays was also assessed by testing positive controls and clinical specimens. As shown in Figure 2, both assays were able to detect all target viruses and no cross signals were observed. In order to further examine the performance of rMLA assay for mixed infection or co-infection specimens, two mixtures prepared randomly with positive controls, i.e. PC-Mix1 and PC-Mix2, containing three targets respectively, were tested in this study and each target was detected successfully with no interference signals from the others. The results showed that the rMLA had satisfying analytical specificities and multiplex detection capacity for target viruses.

### Analysis of diagnostic performance

The new-developed rMLA and two-panel multiplex real-time RT-PCR were assessed for their diagnostic performance in practical application through detecting clinical specimens. The diagnostic sensitivities and specificities of both for each viral target were determined using the results of laboratory diagnosis by in-house monoplex real-time RT-PCR as the comparator with software SPSS version 19.0 (Table 1). No data were available for MERS-CoV due to the lack of positive specimens. Results showed that the rMLA assay has good diagnostic sensitivities for FluA (97.10%, *n* = 293), FluB (94.59%, *n* = 157), PIV3 (98.68%, *n* = 133), RSV (94.87%, *n* = 74) and MPV (95.92%, *n* = 82), approximate to the multiplex real-time RT-PCR. In order to observe the diagnostic consistency between the two new-developed assays, both of detection data were re-analyzed by SPSS (Table 2). The Kappa values of five targets were more than 0.919, suggesting a great consistency in their diagnostic performance. In addition, we found that all of false-negative specimens for RSV and MPV by rMLA had real-time PCR results of *C*_T_ over 38. The *C*_T_ values of specimens with false-negative results ranged from 38.1 to 39.6 for RSV; and the values ranged from 38.3 to 39.8 for MPV. This suggested that the detection limits of rMLA for the two targets were lower than the multiplex real-time RT-PCR.

**Table 1:** Diagnostic sensitivity and specificity of the Luminex-based rMLA and the real-time RT-PCR assay^a^

Target	Lab Diagnosis^b^	rMLA assay				Real-time RT-PCR assay^c^			
		+	-	Sensitivity (%)	Specificity (%)	+	-	Sensitivity(%)	Specificity(%)
FluA	+	234	7	97.10	96.15	232	9	96.27	96.15
	-	2	50			2	50		
FluB	+	105	6	94.59	100.00	104	7	93.69	100.00
	-	0	46			0	46		
PIV3	+	75	1	98.68	96.49	73	3	96.05	98.25
	-	2	55			1	56		
RSV	+	37	2	94.87	97.14	35	4	89.74	94.29
	-	1	34			2	33		
MPV	+	47	2	95.92	100.00	45	4	91.84	96.97
	-	0	33			1	32		

^a^Plus mark, Positive; minus mark, Negative. ^b^Laboratory diagnostic methods used previously, adopting in-house monoplex real-time RT-PCR. ^c^Representing the two-panel multiplex real-time RT-PCR assay developed in this study.

**Table 2:** Consistency in diagnostic performence of the rMLA and real-time RT-PCR assay^a^

Target	Real-time RT-PCR^b^	rMLA		Accordance Rate(%)	Kappa	Approx. Sig.
		+	-			
FluA	+	234	0	99.32	0.979	0.000
	-	2	57			
FluB	+	104	0	99.36	0.986	0.000
	-	1	52			
PIV3	+	74	1	97.74	0.954	0.000
	-	2	56			
RSV	+	36	1	95.95	0.919	0.000
	-	2	35			
MPV	+	45	1	96.34	0.925	0.000
	-	2	34			

^a^Plus mark, Positive; minus mark, Negative. ^b^Representing the two-panel multiplex real-time RT-PCR assay developed in this study.

### Analysis of assay reproducibility

In order to learn the performances of assays in the hands of others, the reproducibilities of developed rMLA and multiplex real-time RT-PCR assay were assessed. Same positive specimen for each target was tested in duplicate by three different technicians for ten times in three months to monitor the inter-assay variation. That for MERS-CoV due to the lack of positive specimen was replaced by a positive control with 10^5^ copies/μl of RNA transcripts. The coefficient of variations (CVs, *n* = 10) of inter-assay were fluA 9.68%, fluB 7.22%, PIV3 13.48%, RSV 8.81%, MPV 10.32%, MERS-CoV 9.17% for the rMLA, and fluA 9.03%, fluB 8.16%, PIV3 12.12%, RSV 8.54%, MPV 9.62%, MERS-CoV 8.29% for the real-time RT-PCR. The CVs of intra-assay obtained by testing ten positive specimens in duplicate for each target(using positive controls for MERS-CoV), were fluA 6.21%, fluB 5.20%, PIV3 8.76%, RSV 7.28%, MPV 7.68%, MERS-CoV 6.41% for the rMLA, and fluA 7.04%, fluB 7.52%, PIV3 9.83%, RSV 8.53%, MPV 8.40%, MERS-CoV 6.06% for the real-time RT-PCR. The results showed that the reproducibilities of two assays are comparable in practical application.

### Analysis of cost effectiveness

Reagent cost and turnaround time of our developed rMLA and two-panel multiplex real-time RT-PCR were listed in Table 3. The real-time RT-PCR with the advantages of simultaneous target amplification and analysis, required ∼2 h (130 min) to test one plate if two panels of the assay were availably setup in one 96-well plate (the number of tested specimens is less than 46, due to at least 2 wells needed for positive and blank control). While, the rMLA took at least ∼3 h (190 min) to test one plate (<= 93 specimens, 3 wells for positive PC-Mix1, PC-Mix2 and blank control) due to the subsequent hybridization and Luminex analysis after target amplification.

**Table 3:** Cost-effectiveness comparison of the rMLA and real-time RT-PCR assay

Assays	Time/plate	Time (2 plates)	Cost/reaction^a^	Cost(2 plates)^a^
Real-time RT-PCR^b^				
RNA extraction	40 min	80 min	$6.25	$1,200.00
First RT-PCR Panel 1 (25 μl) and analysis	90 min	90 min	$3.31	$318.00
TaKaRa One Step RT-PCR reagents			$0.78	
Primers (forward and reverse, 3 targets)			$0.19	
TaqMan fluorescent probes (3 targets)			$2.34	
First RT-PCR Panel 2 (25 μl) and analysis		90 min	$3.31	$318.00
Second RT-PCR Panel 1 (25 μl) and analysis		90 min		$318.00
Second RT-PCR Panel 2 (25 μl) and analysis		90 min		$318.00
Total	130 min	440 min	$12.88	$2,472.00
rMLA				
RNA extraction	40 min	80 min	$6.25	$1,200.00
RT-PCR (25 μl volume)	90 min	90 min	$1.72	$330.00
TaKaRa One Step RT-PCR reagents			$0.78	
Primers (biotin-forward and reverse, 6 targets)			$0.94	
Luminex analysis (hybridization &.Reading data)	60 min	90 min		
Capture probes (6 targets) &. hybridizatio reagents			$5.47	$1,050.00
Total	190 min	260 min	$13.44	$2,580.00

^a^Based on the purchase prices in China in 2015 and the condition of an ordinary lab with only one standard PCR, a real-time PCR and a Luminex instrument. These do not include costs for common lab reagents, disposables, instruments and labor.

^b^Representing the two-panel multiplex real-time RT-PCR assay developed in this study.

A workload of two plates (i.e. 2*96 reactions) were used to compare the multi-target and throughput capability of two assays . The two-panel multiplex real-time RT-PCR needed four times to spend up to ∼7 h (440 min) and $2,472 ($12.88/reaction) for all reactions when only one real-time PCR instrument was available; While, the Luminex-based rMLA need at most 6 h (350 min) and $2,580 ($13.44/reaction). If an ordinary/standard thermocycler was simultaneously available for amplification, being feasible in most labs, the overall turnaround time of the rMLA assay could be shorten to ∼4.5 h (260 min). If more specimens needed to be tested within an 8-h clinical shift, the rMLA can still be setup by one technician to run another plate with another ordinary thermocycler. This is an easier and more cost-effective alternative to purchase and run several real-time instruments in parallel. Additionally, above cost analysis was based on the reagent prices in China, which are obviously higher than those in US, especially the Luminex beads. It was reported that the total reagent cost for a Luminex xMAP analysis was $3.90 per test, and $9.42 per test for a three-panel duplex real-time RT-PCR to detect six targets [20].

## DISCUSSION

In this study, we developed two multiplex assays: a Luminex-based rMLA and a two-panel triplex real-time RT-PCR for six respiratory viruses, five of which were common respiratory viruses in Jiaxing, i.e. FluA, FluB, PIV-3, RSV, and MPV. While the MERS-CoV is a potentially threatening virus to Jiaxing. This is the first report to describe a Luminex-based laboratory-developed assay designed specifically in order to include MERS-CoV as one of targets. Several commercial Luminex-based kits for respiratory viruses are available now, such as xTAG^™^ respiratory virus panel (RVP) and xTAG^™^ RVP Fast (Luminex Corp.), MultiCode^®^-PLx (EraGen Biosciences), ResPlex II Kit (QIAGEN) [26, 32, 33], however , they either involve too complicated steps, or include too many targets , or do not have targets we need, e.g. MERS-CoV. Importantly, they are too expensive for routine detection in clinical laboratory.

Over the past ten years, ‘in-house’ real-time PCR/RT-PCR assays have been used by our lab for the diagnosis and surveillance of viral respiratory tract illnesses and showed the good characteristics of rapid and simplicity due to the advantage of simultaneous amplification and analysis, without post-amplification manipulation [34, 35]. However, the primers and probes provided by provincial CDC and state CDC were designed specifically for individual target, so these ‘in-house’ assays for lab diagnosis were mostly monoplex. Although multiplex real-time RT-PCR was also used in work, the limited multiplexing and throughput capacity became its obvious shortcomings due to the limited fluorescent channels in a real-time instrument and the adverse interactions between primers and probes in one reaction [20, 35]. So, a multiplex real-time RT-PCR assay for more targets has to be divided into two or more panels, such as our developed real-time RT-PCR assay with two panels for six respiratory viruses in this study. This makes the limited throughput capacity more detrimental and tight. In epidemic period, the assay requires more plates, more technicians and more turnaround time, otherwise the lab needs to buy more machines in parallel to run a large number of samples within a limited work shift.

In view of the facts, the rMLA assay was developed on a Luminex multiplex platform (Bio-Plex 200) for multi-target and high-throughput detection. The platform allows for detecting more targets simultaneously and performing continuous analysis for plate queue. The bottleneck of multiplex PCR in Luminex xMAP technology for nucleotide analysis, though still exists, is not so intractable due to the post-amplification analysis. This decreases the possibility of interferences from capture probes in that they are added in the hybridization step. And the primer/probe design for a Luminex assay becomes easier because the criterion of probe and the size of amplicon are not as strict as a real-time PCR. However, the product should be less than 300 bp to assure the amplification efficiency and analytical sensitivity [36]. In this study, the same primers and probes (slightly modified) shared by the rMLA and the real-time RT-PCR ensured not only the amplification efficiency and sensitivity of the rMLA assay, but also ensured the comparability of the two assays. Actually we had explored the possibility of transplanting a real-time PCR assay to a Luminex xMAP analysis. Our results indicated that this is feasible, of course, and may need slight modifications specially for the Luminex analysis.

In this study, our developed rMLA assay and multiplex real-time PCR assay had diagnostic sensitivities of more than 90% for the five targets (No Data for MERS-CoV due to no positive specimens) and no cross reaction was found. No obvious difference was also found between the both, and in fact the great consistency of the diagnostic performance presented in the detection of clinical specimens(accordance rates > 95%, Kappa values > 0.9 for all targets). Some reports had the similar result [36, 37], but there were also reports that Luminex xMAP analysis for some target was more or less sensitive than real-time PCR [20, 37]. This mostly depended on the primer design and reaction optimization of the assay according to our experiences. Sensitivity analysis showed that the detection limits of the rMLA for FluA, FluB, PIV-3 and MERS-CoV were 5–25 copies/μl of viral RNA, approximate to the real-time RT-PCR, and those of the rMLA for MPV and RSV were 8 and 22copies/μl, lower than the real-time RT-PCR(78 and 114 copies/μl respectively). But, the detection limits of the rMLA for the six targets could be up to 6, 9, 5, 15, 6 and 10 copies/μl, if the cutoffs for rMLA were set at three times the blank control recommended by ACS Commitee on Environmental Improvement [38]. Above results indicated that the overall performance of rMLA was comparable to the real-time RT-PCR, suggesting that the analytical sensitivities of rMLA for two targets, RSV and MPV, may be higher than the real-time RT-PCR, despite not confirmed by our statistical data in diagnosis.

Technically, Luminex xMAP analysis has higher sensitivity and specificity than real-time PCR since the liquid-phase hybridization after amplification can decrease the background interference and two-fluorescence detection can increase the specificity [28, 36]. Through the study, we believe that we can make the rMLA assay better with some improvements. Its sensitivity and specificity can be improved by asymmetric multiplex PCR, adopting a protocol of differential cycle conditions based on temperature differential primer design [20, 39, 40]. The extra exonuclease I can be added to eliminate the residual primers before hybridization [11, 36]. So, the overall turnaround time of the assay may increase a little (less than 30 minutes). In addition, the reaction volume of amplification will be adjusted to 10 μl (containing 2.5 μl of viral RNA template), and all product will be used for hybridization so that the amplification, hybridization and analysis of the rMLA can be all handled in only one 96-well plate. This will make the assay more convenient and economic, and greatly reduce the probability of carryover contamination between pre- and post-amplification steps. However, these could be our next work. Though we have not done so yet, a large number of data based on the established research program had been obtained and were in line with our study objectives.

In conclusion, the new-developed real-time RT-PCR for 6 common respiratory viruses is suitable for the emergency detection of a small number of specimens due to the advantage of rapid and simplicity. While, our developed Luminex-based rMLA has great sensitivity and specificity comparable to the real-time RT-PCR, and a higher throughput capability to run large numbers of specimens simultaneously, significantly reducing the cost. Furthermore, due to the Luminex platform’s flexibility, the rMLA can be expanded to include more respiratory pathogen targets, to meet local seasonal or emerging changes of respiratory infectious diseases.

## MATERIALS AND METHODS

### Ethics statement

Informed consent was obtained from all participants before this study was conducted. The study and all procedures were approved by Jiaxing Municipal Center for Disease Control and Prevention (Jiaxing CDC), and carried out in accordance with biosafety and ethical standards of the institutional and national research committee and the relevant laws and regulations of People’s Republic of China.

### Clinical specimens and positive controls

A total of 739 respiratory specimens were mainly from specimens submitted to Jiaxing CDC by hospitals in Jiaxing from 2013 to 2015, and partially from Zhejiang Provincial Center for Disease Control and Prevention (Zhejiang CDC). These specimens were mostly nasopharyngeal swabs, a small number of tracheal aspirations and bronchoalveolar lavages, and were collected in viral transport medium and stored at −80°C. All had been tested by in-house real-time RT-PCR (monoplex) according to the relevant diagnostic criteria of respiratory tract infection. The used primers and probes were provided by Zhejiang CDC and Chinese CDC, or synthesized in accordance with the sequences from both.

Additionally, all positive controls of target viruses (Table 4) were prepared by synthesizing target DNA and transcribing it into RNA. Target DNAs were synthesized by Shanghai Sangon Biotech Co., Ltd. (Sangon) and RNA transcription was performed with the MEGAscript^®^ RNAi Kit (Catalog#AM1626, Ambion, USA). After purification and concentration using MagMAX^™^-96 Viral RNA Isolation Kit (Catalog#AM1836, Ambion, USA) on KingFisher Flex system (Thermo Fisher Scientific Inc., USA), the *in vitro* RNA transcripts were quantified with NanoDrop^™^ 2000 microspectrophotometer (Thermo Fisher Scientific Inc., USA) and adjusted to 10^7^ copies per μl using EASY Dilution for Real-Time PCR (Cat#D9160A, TaKaRa, China,Dalian) as original quantification standards in this study. The dilutions containing 10^5^ copies/μl of viral RNA transcripts were used as positive controls and 10-fold serial dilutions were used to determine analytical sensitivity of the following assays. All of these were stored at −80°C until analysis.

**Table 4:** Positive controls used in this study

Positive control	Sequence(5′–3′)	Reference seq ID and position^a^
FluA-PC	GAAAGAACACAGATCTTGAGGCTCTCA TGGAATGGCTAAAGACAAGACCA ATCTTGTCACCTCTGACTAAGGGA ATTTTAGGATTTGTGTTCACGCTCA CCGTGCCCAGTGAGCGAGGACTGC AGCGTAGACGATTTGTCCAAAATGC CCTAAATGGGAATGGGGACCCGAACAA CATGGATAGAGCAGTTAAACTATAC	KP317439:101-300 Influenza A virus (A/Delhi/053/2011(H1N1)), matrix protein 1 (M1) gene, complete cds.
FluB-PC	TGGAGGATGAAGAAGATGGCCATCGGAT CCTCAACTCACTCTTCGAGCGTCTTAATG AAGGACATTCAAAGCCAATTCGAGCAGC TGAAACTGCGGTGGGAGTCTTATCCCAAT TTGGTCAAGAGCACCGATTATCAC CAGAAGAGGGAGACAA	KT223814:707-860 Influenza B virus (B/California/NHRC_M1023/2014) segment 8 nuclear export protein (NEP) gene, partial cds and nonstructural protein 1 (NS1) gene, complete cds.
PIV3-PC	CACAGGAAGCATTGTATCATCTGT CATATTGGACTCACAAAAATCGA GAGTCAACCCAGTCATAACTTACT CAACAGCAACCGAAAGGGTA AACGAGCTGGCTATCCGAAACAAAACACT	KJ672618:8181-8300 Human parainfluenza virus 3 strain HPIV3/Homo sapiens/PER/CFI1849/2012, complete genome.
RSV-PC	TGGGGCAAATATGGAAACATACG TGAACAAACTTCACGAGGGCTC CACATACACAGCTGCTGTTCA ATACAATGTCCTAGAAAAAGA CGATGATCCTGCATCACTTACA ATATGGGTGCCCATGTTCCAA	KP317953:3251-3380 Human respiratory syncytial virus isolate Kilifi_11862_29_RSVA_2011, complete genome.
MPV-PC	ATGTCTCTTCAAGGGATTCAC CTAAGTGATCTGTCATATAAA CATGCCATATTAAAAGAGTCT CAATACACAATAAAAAGAGAT GTAGGCACCACAACTGCAGTG ACACCTTCATCATTGCAACAAG AAATAACACTT	KJ627435:1-138 Human metapneumovirus strain HMPV/Homo sapiens/PER/FLE0425/2009/A, complete genome.
mrsCoV-PC	CCACTGTTTTCGTGCCTG CAACGCGCGATTCAGTTCCTCTT CACATAATCGCCCCGAGCTCGCT TATCGTTTAAGCAGCTCTGCGCTA CTATGGGTCCCGTGTAGAGGCTAA TCCATTAGTCTCTCTTTG	KP209307:27441-27570 Middle East respiratory syndrome coronavirus strain Abu Dhabi_UAE_18_2014, complete genome.

^a^Reference seq ID is accession No. of the sequence in NCBI GenBank.

### Viral RNA extraction

All specimens were vortexed and resuspended before viral RNA extraction. 75 μl specimens were used to extract viral RNA by using the MagMax^™^-96 Viral RNA Isolation Kit on the KingFisher Flex system according to the manufacturer’s recommendation. 75 μl viral RNA extracts were collected and stored at −80°C before use. In addition, FluA positive control (FluA-PC), containing 10^5^ copies/μl of FluA RNA transcripts, was added into extraction procedure in order to monitor RNA extraction efficiency by comparing the concentrations before and after the extraction.

### Primers and probes

The primers, TaqMan probes and capture probes used in this study were listed in Table 5. Those aiming at FluA, PIV3, RSV, MPV were improved or redesigned by our lab, so that the targets of the assays included new emerging or more viruses, such as FluA target including influenza A virus subtype 2009 pdm H1N1 and H7N9 2013, RSV target including RSV group A and B. Primers and TaqMan probes for FluB and MERS-CoV were not changed, respectively from the sequences recommended by Chinese National Influenza Center (CNIC) and World Health Organization (WHO). All of the forward primers, reverse primers, TaqMan probes and modified forward primers(-F+) with 5′-biotin were synthesized by Sangon, and the capture probes(-P+) coupled to fluorescent-encoding microspheres (beads) through 5′ amino-C12 linker, only used for Luminex-based rMLA assay, were designed by our lab and synthesized by Shanghai Tellgen Life Science Co., Ltd. (Tellgen).

**Table 5:** PCR primers, Taqman probes and capture probes used in this study

Target	Primer or Probe^a^	Sequence(5′-3′)^b^	Reference seq and position^c^	Modification	Reaction Concentration (µM)
FluA Matrix protein(M) gene	FluA-F/FluA-F+	GACCRATCYTGTCACCTCTGAC	KP317439:146-167	FluA-F+:5′-Biotin	0.5
	FluA-R	GGGCATTYTGGACAAAKCGTCTACG	KP317439:226-250		0.5
	FluA-P	TGCAGTCCTCGCTCACTGGGCACG	KP317439:201-224(-)	5′-FAM,3′-BHQ1	0.25
	FluA-P+	AGTCCTCGCTCACTGGGCAC	KP317439:204-223(-)	5′-linker-bead(#34)	0.25
FluB Nuclear Export Protein (NEP) gene	FluB-F/FluB-F+	TCCTCAACTCACTCTTCGAGCG	KT223814:734-755	FluB-F+:5′-Biotin	0.5
	FluB-R	CGGTGCTCTTGACCAAATTGG	KT223814:816-836		0.5
	FluB-P	CCAATTCGAGCAGCTGAAACTGCGGTG	KT223814:778-804	5′-HEX,3′-BHQ1	0.25
	FluB-P+	CCAATTCGAGCAGCTGAAACTG	KT223814:778-799	5′-linker-bead(#42)	0.25
PIV3 Nucleoprotein(N) gene	PIV3-F/PIV3-F+	GGAAGCATTGTRTCATCTGTC	KJ672618:8185-8205	PIV3-F+:5′-Biotin	0.6
	PIV3-R	TCGGATRGCCAGCTCGT	KJ672618:8273-8289(-)		0.6
	PIV3-p	ACCCAGTMATAACTTACTCAACAGC	KJ672618:8234-8258	5′-ROX,3′-BHQ1	0.3
	PIV3-p+	ACCCAGTMATAACTTACTCAACA	KJ672618:8234-8256	5′-linker-bead(#21)	0.3
RSV Matrix protein(M) gene	RSV-F/RSV-F+	GCAAATATGGAAACATACGTGAACA	KP317953:3255-3279	RSV-F+:5′-Biotin	0.5
	RSV-R	GCACCCATATTGTWAGTGATGCA	KP317953:3347-3369(-)		0.5
	RSV-P	CTTCACGAGGGCTCCACATACACAGC	KP317953:3282-3307	5′-FAM,3′-BHQ1	0.25
	RSV-P+	AGGGCTCCACATACACAGC	KP317953:3289-3307	5′-linker-bead(#26)	0.25
MPV Nucleoprotein(N) gene	MPV-F/MPV-F+	TCTCTTCAAGGGATTCACCT	KJ627435:4-23	MPV-F+:5′-Biotin	0.48
	MPV-R	GTTATTTCTTGTTGCAATGATGA	KJ627435:112-134(-)		0.48
	MPV-P	CATGCYATATTAAAAGAGTCTCARTAC	KJ627435:43-69	5′-HEX,3′-BHQ1	0.24
	MPV-P+	CATGCYATATTAAAAGAGTCTCA	KJ627435:43-69	5′-linker-bead(#48)	0.24
MERS-CoV upstream of E protein(upE) region	mrsCoV-F/mrsCoV-F+	GCAACGCGCGATTCAGTT	KP209307:27458-27475	mrsCoV-F+:5′-Biotin	0.5
	mrsCoV-R	GCCTCTACACGGGACCCATA	KP209307:27530-27549(-)		0.5
	mrsCoV-P	CTCTTCACATAATCGCCCCGAGCTCG	KP209307:27477-27502	5′-ROX,3′-BHQ1	0.25
	mrsCoV-P+	CTCTTCACATAATCGCCCCGAGC	KP209307:27477-27497	5′-linker-bead(#62)	0.25

^a^F,R,P respectively represents forward primer, reverse primer and TaqMan probe used for real-time RT-PCR. Modified forward primer(F+) and capture probe(P+),with plus mark, only used for Luminex-based rMLA.^b^Degenerate bases: R,A or G; Y,C or T; K, T or G; M, A or C; W, A or T. ^c^Reference seq ID is accession number of the sequence in NCBI GenBank. Sequence with minus mark is antisense.

### Real-time RT-PCR assay

A one step multiplex real-time RT-PCR assay was developed by our lab using TaKaRa One Step PrimeScript™ RT-PCR Kit(Cat# RR064A), and divided into Panel 1 for FluA, FluB, PIV3 and Panel 2 for RSV, MPV and MERS-CoV, due to only 5 channels available for detection on the CFX96^™^ Touch Real-time PCR System(Bio-Rad Laboratories Inc., USA). The final 25 μl mixture for each reaction contained 5μl viral RNA extract and optimized concentrations of the primers (-F and -R) and TaqMan probes (Table 2). Both panels of the real-time RT-PCR assay were performed in condition of 42°C for 30min, 95°C for 1min, and 45 cycles of 95°C for 15sec and 55°C for 45sec. Qualitative result of the assay was determined as follows: the threshold cycle value (*C*_*T*_) < 35, positive; no amplification or *C*_*T*_ > 38 (considered as an invalid amplification), negative; *C*_*T*_ between 35–38, reserved, if still range between 35–38 after redoing, considered it as positive. The average *C*_*T*_ value was determined for each standard dilution or sample in two replicates, and the detection limit of the assay for each target was estimated from the standard curve at a cutoff point of *C*_*T*_ value (The value was set to 38 in this study).

### Luminex-based rMLA assay

A multiplex RT-PCR for 6 targets, including FluA, FluB, PIV3, RSV, MPV and MERS-CoV, was performed with the same TaKaRa One Step PrimeScript^™^ RT-PCR Kit. The 25 μl reaction mixture contained 5 μl viral RNA extract and optimized concentrations of biotin-modified forward primers (-F+) and reverse primers (-R) (Table 5), using same sequences as those of real-time RT-PCR assay described above. After the amplification, 3 μl biotin-labeled PCR product of each reaction was added into 22 μl working bead mixture prepared temporarily (containing 3,000 beads coupled with capture probe of each target) and followed by hybridization of 95°C for 5 min and 50°C for 20 min on the CFX96™ Touch Real-time PCR System. Then, 75 μl of working reporter solution (3 μg/ml streptavidin-phycoerythrin) was added into the mixture followed by incubation at 50°C for 5 min. The original bead solution of each target, original SA-PE reporter solution and 1× tetramethyl ammonium chloride (TMAC) hybridization buffer for dilution were all provided by Tellgen. In the end, 100 μl final volume of the mixture was analyzed on a Bio-Plex 200 system (Bio-Rad Laboratories Inc., USA) at 48°C. The median fluorescence intensity (MFI) of at least 50 beads was reported for each bead set. The average MFI value was determined for each standard dilution or sample in duplicate. The cutoff value for a positive result was set at five times the background MFI value of the blank control and the detection limit of the assay for each target was calculated from the standard curve at the cutoff of MFI value.
